# Assessing the Reliability of D-Dimer Measurement in EDTA Plasma: A Comparison to the Established Citrate Method

**DOI:** 10.3390/diagnostics15131720

**Published:** 2025-07-06

**Authors:** Daniel Pfingst, Adriana Méndez, Peter Neyer, Henning Nilius, Nicole Schaub, Patricia Keusch, Michael Nagler, Angelika Hammerer-Lercher

**Affiliations:** 1 Institute of Laboratory Medicine, Cantonal Hospital Aarau, 5001 Aarau, Switzerland; daniel.pfingst@ksa.ch (D.P.); adriana.mendez@ksa.ch (A.M.); peter.neyer@ksa.ch (P.N.); nicole.schaub@ksa.ch (N.S.); patricia.keusch@ksa.ch (P.K.); 2 Department of Clinical Chemistry, Inselspital, Bern University Hospital, University of Bern, 3010 Bern, Switzerland; henning.nilius@insel.ch (H.N.); michael.nagler@insel.ch (M.N.)

**Keywords:** D-dimer, luminescent oxygen channeling assay (LOCI), hemostasis, EDTA plasma

## Abstract

**Background:** D-dimer determined in citrated plasma is a well-established and efficient biomarker, particularly for ruling out venous thromboembolism. In certain clinical settings, the availability of citrated plasma may pose challenges when not readily available. To address this issue, we investigated the feasibility of using ethylenediaminetetraacetic acid (EDTA) plasma as an alternative specimen for D-dimer measurement. **Methods:** Our study evaluated anonymized plasma samples (n = 99, for both citrate and EDTA) using the INNOVANCE^®^ D-dimer assay, an automated particle-enhanced immunoassay, and the INNOVANCE^®^ LOCI hs D-dimer assay, leveraging the luminescent oxygen channeling assay (LOCI) method. **Results:** The assays demonstrated a correlation of r ≥ 0.97 (95% CI 0.96 to 0.98) within citrated plasma and maintained a similar correlation r ≥ 0.96 (95% CI 0.94 to 0.97) between citrate and EDTA plasma upon correction for the dilution effect of the sodium citrate solution. **Conclusions**: These results indicate that the utilization of EDTA instead of citrate plasma is feasible and may provide similar diagnostic information. However, the observed variance could have an impact on clinical interpretation and risk assessment. Therefore, future studies are needed to confirm the results and, if necessary, determine cut-off values and clinical performance.

## 1. Introduction

The measurement of D-dimer, along with clinical information, has a major impact on triage and treatment of patients with deep vein thromboses or pulmonary embolisms [1,2]. In accordance with current guidelines, measurement with a high-sensitivity D-dimer assay is a cost- and time-efficient standard test.

In low-risk patients, a negative result for this coagulation biomarker allows ruling out venous thromboembolism and could save further expensive diagnostic imaging. However, D-dimer values of patients aged 50 years or older, post-operative patients, or pregnant women may be regarded as elevated when assessed against commonly used thresholds. Positive D-dimer results are frequently observed in these groups without a clinical correlation, contributing to the low specificity of this parameter. This must be taken into account in clinical interpretation and risk assessment. As soon as there is a high risk of venous thromboembolism, imaging is mandatory [3]. In addition, D-dimer assessment is a valuable and representative laboratory parameter to estimate the activity of recent or ongoing coagulation and fibrinolytic activity [4].

Over nearly five decades, various techniques and assays to measure fibrin degradation products have been developed, evaluated, and implemented [5]. There are major differences in the concordance of different routine assays, depending on the area of application, such as point-of-care or laboratory routine, as well as depending on the specimen and test method [6]. In the past, enzyme-linked fluorescence immunoassays (ELFAs), enzyme-linked microplate immunosorbent assays (ELISAs), and quantitative latex-enhanced turbidimetric immunoassays have demonstrated high diagnostic sensitivity but moderate specificity [7].

However, enough citrate plasma may not always be readily available in the laboratory for D-dimer measurement. This is particularly true when clinicians issue additional orders following an initial laboratory request, potentially necessitating an extra blood sample. In urgent situations, which may occur in emergency departments, where time is crucial, the available volume of citrate plasma may be limited, particularly in infants. Moreover, underfilling of blood tubes in these high-pressure situations can result in a rejection of coagulation measurements altogether.

To overcome this possible drawback, we investigated the reliability of D-dimer determination in ethylenediaminetetraacetic acid (EDTA) plasma, applying different methods in comparison to the traditional citrate plasma measurement. It is known that functionally measured hemostasis parameters, depending on clot formation, may be affected when measured in EDTA plasma. Prolonged PT and APTT times, as well as reduced coagulation factors, may distort the interpretation regarding inhibitors. [8,9]. By contrast, there are some D-dimer assays available and validated also for running with EDTA plasma. However, these assays are predominantly point-of-care assays [6].

We aimed to investigate the performance of D-dimer in EDTA plasma compared to citrate plasma using two different methods in a single-center method-comparison quality study.

## 2. Methods

### 2.1. Study Design, Setting, and Population

We conducted a single-center method-comparison quality study at the Cantonal Hospital Aarau (KSA) using fully anonymized samples, not related to any health care records. The following inclusion criteria were applied: (a) current D-dimer measurement ordered, (b) citrate and EDTA plasma samples readily available in the laboratory, (c) enough sample material, (d) age > 18 years, and (e) signed general informed consent.

Exclusion criteria were (a) preanalytical issues such as hemolytic, bilirubinemic, and lipemic samples, (b) not enough sample material, and (c) samples with results of <0.2 mg/L fibrinogen equivalent units (FEU) using the contemporary INNOVANCE^®^ D-dimer assay were excluded to avoid missing values for comparison.

### 2.2. Sample Selection

We aimed to include 50 to 100 sample pairs in accordance with generally accepted method comparison guidelines such as CLSI. The plasma samples were selected to cover the entire initial analytical measurement range of the routinely used D-dimer assay. However, we also placed particular consideration on the manufacturer’s recommended cut-off value of 0.5 mg/L FEU, because this reflects the established cut-off in clinical practice for the triage of thromboembolic events. Therefore, using the contemporary method, samples near the cut-off (n = 30) in the range of 0.4–0.6 mg/L FEU were collected, and the remaining samples (n = 69) were chosen equally from the upper and lower measurement range.

### 2.3. Handling of Samples

Deidentified remnants of freshly collected citrate and EDTA anticoagulated plasma samples (n = 99 for both citrate and EDTA), received for routine laboratory determinations at the Institute of Laboratory Medicine of the Cantonal Hospital Aarau (KSA), were used for quality assurance and quality control to investigate the usability of EDTA specimen for D-dimer determination. Standardized plastic Vacutainer tubes (Becton Dickinson, Allschwil, Switzerland) used for blood sampling contained either sodium citrate solution (0.109 mol/L 3.2%) or K_2_EDTA (size-dependent, either 3.6 mg for 2 mL or 7.2 mg for 4 mL, spray-dried in the tube). Plasma was obtained within 2 h of collection by centrifugation at 3000× *g* for 5 min at 18 °C.

### 2.4. Correction for Citrate Dilution

EDTA is spray-dried in the sample tube, whereas citrate tubes contain liquid sodium citrate solution as an anticoagulant, and the addition of blood results in a mixing ratio of one part citrate solution to nine parts blood in the tube. To correct for this dilution effect, the results measured in EDTA plasma were therefore multiplied by a factor of 0.9 for method comparison.

### 2.5. D-Dimer Determination

For D-dimer measurement, the contemporary assay was routinely applied in citrate plasma as usual and complemented by the LOCI high sensitivity (hs) D-dimer assay.

Subsequently, we performed measurements of EDTA samples with both assays for the method comparison. The samples were measured (between September 2021 and February 2022) on the Atellica^®^ COAG 360 (both assays, as well as the analyzer, are from Siemens Healthineers, Marburg, Germany).

### 2.6. Assay Specifications

#### 2.6.1. INNOVANCE^®^ D-Dimer Assay

The contemporary D-dimer assay is an automated particle-enhanced immunoassay, using polystyrene particles coated with monoclonal mouse antibodies that bind to cross-linked fibrin degradation products (D-dimer) in a targeted manner and with high affinity. Samples containing D-dimer lead to an increase in turbidity, which is detected photometrically between 560 and 575 nm. The results are provided in mg/L fibrinogen equivalent units (FEU). FEU expresses the concentration of fibrin degradation products in relation to the gravimetrically determined mass of fibrinogen from which they were derived [10,11].

#### 2.6.2. INNOVANCE^®^ LOCI hs D-Dimer Assay

The hs D-dimer is an automated immunoassay utilizing the luminescent oxygen channeling assay (LOCI) method. The LOCI technology enables the detection of extremely low quantities of analyte and facilitates real-time measurement of particle binding kinetics [12]. The assay uses the binding of two synthetic beads. A chemibead coated with a primary anti-D-dimer monoclonal antibody and a chemiluminescent dye, as well as a sensibead coated with streptavidin and a light-sensitive dye. A biotinylated, secondary anti-D dimer monoclonal antibody is subsequently added and binds to the streptavidin-coated sensibead. Adding sample material containing the analyte of interest brings both beads into spatial proximity, in proportion to the analyte’s abundance. Excitation of the sample with light at 680 nm results in the release of singlet oxygen from the photosensitive dye bound to the sensibead. If there is no spatial proximity of chemibead and sensibead, the singlet oxygen dissociates before it can trigger further luminescent reactions. Only where beads have bound to an analyte, they come into spatial proximity close enough to allow a chemiluminescent reaction to occur, which is then measured with a luminometer. The luminescence emission is detected at 612 nm and allows determination of the concentration of the analyte [13]. Thus, interferences and non-specific binding are reduced, resulting in an excellent signal-to-noise ratio that allows measurements of very low concentrations.

### 2.7. Precision Assessment

For precision assessment, low (~0.25 mg/L FEU) and high (~1.4 mg/L FEU) control plasma pools were prepared from remnants of freshly collected plasma samples and shock frozen in aliquots at −80 °C. Measurements were performed, in accordance with the CLSI EP15 guidelines, five times per run and over a period of five days with each method.

### 2.8. Statistical Analysis

We assessed the performance of D-dimer in EDTA compared to citrate plasma samples, as well as the performance of the two different analysis methods by Passing–Bablok regression and Bland–Altman difference plots. Correlations were analyzed by Spearman’s rank correlation coefficient. The Kolmogorov–Smirnov test was performed for normal distribution. For group comparison, an independent samples t-test was used for normally distributed data categories. A *p*-value below 0.05 was considered statistically significant. The stated statistics were performed using MedCalc statistical software version 19.6 (MedCalc Software Ltd., Ostend, Belgium). Repeatability and within-laboratory standard deviations were performed by a calculated multifactorial analysis of variance (with R version 4.0.5 (The R Foundation for Statistical Computing, Vienna, Austria)).

## 3. Results

### 3.1. Method Comparison

#### 3.1.1. Precision

The coefficients of variation (CVs) were <5% for the contemporary assay and <1.5% for the hs LOCI method in citrate plasma (Table 1 and Table 2).

#### 3.1.2. Method Comparison

**Figure 1:**
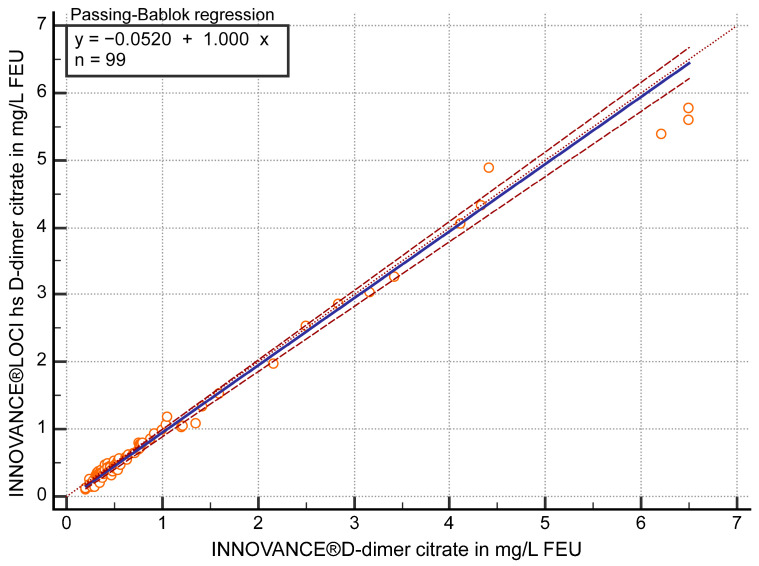
Passing–Bablok regression analysis of D-dimer in mg/L fibrinogen equivalent units (FEU) measured with both methods, the contemporary INNOVANCE^®^ D-dimer and the high sensitivity (hs) D-dimer INNOVANCE^®^ LOCI method in citrate plasma samples (n = 99).

The FEU concentrations in citrate plasma samples (n = 99) measured with both D-dimer methods showed a correlation of r ≥ 0.97 (95% confidence interval (CI) 0.96 to 0.98) and in the Passing–Bablok regression (y = −0.0520 + 1 x) a slope of 1 (95% CI 0.97 to 1.03) (Figure 1). Values of the hs D-dimer assay were approximately 12.5% (95% CI 9.26 to 15.74) lower than those of the current contemporary method (*p* < 0.05) according to Bland–Altman bias calculation.

**Figure 2:**
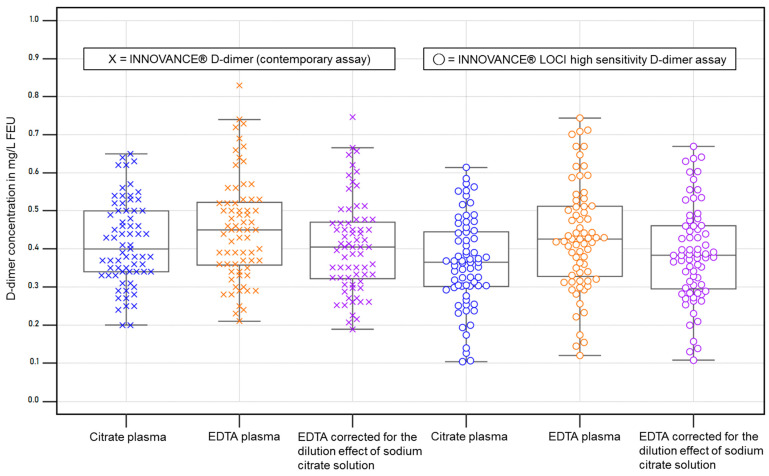
D-dimer concentrations <0.7 mg/L FEU according to the contemporary D-dimer assay in citrate plasma (n = 69) and corresponding plots for each assay and specimen. EDTA corrected: EDTA values multiplied by a factor of 0.9. The boxes represent the values from the 25th to 75th percentile, and the line in the center represents the median. The whiskers extend to the last value before reaching the 1.5 x interquartile range. The dots and crosses depict the individual values.

To examine the performance near the clinical decision value of 0.5 mg/L FEU, only cases <0.7 mg/L FEU (n = 69; Figure 2) according to the contemporary assay were included.

Comparison of both D-dimer methods in citrate plasma samples with FEU concentrations <0.7 mg/L (n = 69) showed a correlation of r ≥ 0.91 (95% CI 0.86 to 0.95) and in the Passing–Bablok regression (y = −0.0746 + 1.05 x) a slope of 1.05 (95% CI 0.94 to 1.16). Values of the hs D-dimer assay were on average 15.7% (95% CI 11.42 to 20.02) lower than those of the current method.

### 3.2. Specimen Comparison

#### 3.2.1. Precision

EDTA plasma samples showed a slightly larger deviation in relation to the precision compared to citrate plasma. The CVs were <6.5% for the routine assay and <2.5% for the hs LOCI method (*p* < 0.05) in EDTA plasma (Table 1 and Table 2).

#### 3.2.2. Performance After Correction for Citrate Dilution

**Figure 3:**
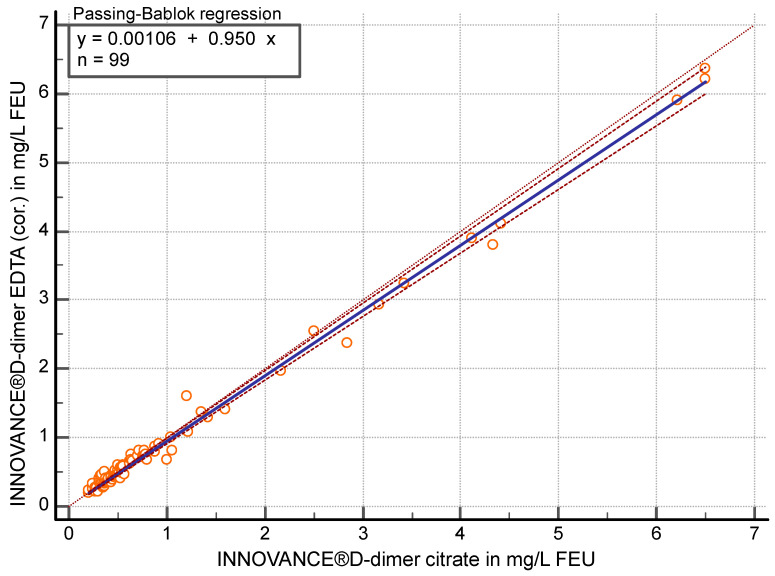
Passing–Bablok regression analysis of D-dimer in mg/L fibrinogen equivalent units (FEU) measured with the contemporary assay for citrate plasma compared to EDTA-corrected (cor.) plasma (n = 99).

After correcting the dilution effect (measured EDTA values were multiplied by 0.9), the correlation was r ≥ 0.96 (95% CI 0.94 to 0.97) between D-dimer in citrate and EDTA-corrected (cor.) plasma (*p* < 0.05) using the contemporary method. Passing–Bablok regression analysis (y = 0.0011 + 0.95 x) showed a slope of 0.95 (95% CI 0.93 to 0.98) (Figure 3) in the comparison of the two specimens. EDTA (cor.) values were on average 3.8% (95% CI 1.16 to 6.42) lower than citrate values.

Comparison of citrate and EDTA (cor.) plasma with FEU concentrations <0.7 mg/L (n = 69) with the contemporary method showed a correlation of r ≥ 0.88 (95% CI 0.81 to 0.92) and in the Passing–Bablok regression (y = −0.0432 + 1.08 x) a slope of 1.08 (95% CI 0.96 to 1.20). EDTA (cor.) values were on average 2.7% (95% CI −0.61 to 5.99) lower than citrate values.

**Figure 4:**
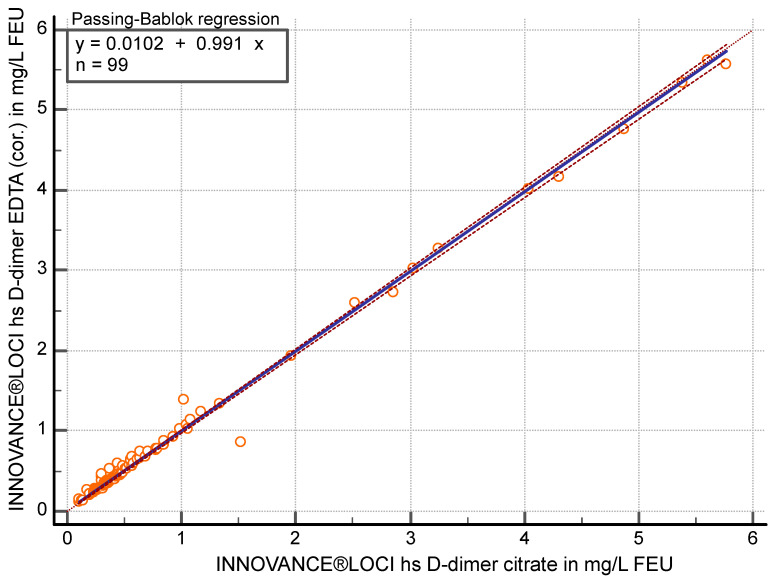
Passing–Bablok regression analysis of D-dimer in mg/L fibrinogen equivalent units (FEU) measured with the high sensitivity (hs) D-dimer assay in citrate plasma compared to EDTA-corrected (cor.) plasma (n = 99).

Concerning the hs-D-dimer method, the correlation was r ≥ 0.97 (95% CI 0.959 to 0.982) between citrate and EDTA (cor.) (*p* < 0.05). In the Passing–Bablok regression (y = 0.0102 + 0.99 x), the slope achieved 0.99 (95% CI 0.98 to 1.00) (Figure 4) with an average of 4.4% (95% CI −6.87 to −1.95) higher values in EDTA (cor.) plasma.

Comparison of citrate and EDTA (cor.) plasma with FEU concentrations <0.7 mg/L (n = 72) with the hs-D-dimer method showed a correlation of r ≥ 0.93 (95% CI 0.89 to 0.96) and, in the Passing–Bablok regression (y = −0.0009 + 1.027 x), a slope of 1.03 (95% CI 0.99 to1.07) with an average of 6.4% (95% CI −9.31 to −3.63) higher values in EDTA (cor.) plasma.

The variance of EDTA samples spread slightly wider than those of citrate plasma samples, and the EDTA values reflect the theoretically expected increases in the correction compared to citrate values (Table 3 and Figure 2).

## 4. Discussion

We assessed the performance of D-dimer measurement, utilizing two different analysis methods in citrate and EDTA anticoagulated plasma samples of 99 patients in a single-center method-comparison quality study.

Considering the entire measurement range, both methods displayed analytically excellent correlation, regression, and mean bias for each specimen, if corrected for the dilution effect of the sodium citrate solution. However, EDTA samples demonstrated a slightly broader distribution compared to citrate samples. Moreover, the performance of the specimen near the clinical decision value of 0.5 mg/L FEU deviates significantly. This is also reflected with regard to precision. Even when using the LOCI method, which is known for its outstanding precision [14] in quantifying a broad range of analytes in different specimens in clinical chemistry, a higher deviation in EDTA plasma (CV < 2.5%) samples was observed compared to citrate plasma (CV < 1.5%) samples.

The accuracy of D-dimer measurement of various tests with high diagnostic sensitivity, but moderate specificity, has been addressed in a vast number of publications [7]. But there are few studies that address the feasibility of D-dimer measurement in EDTA plasma, and those are predominantly used for point-of-care testing [6]. Contrary to functionally measured hemostasis parameters, which depend on clot formation [9] and thus require citrate plasma, D-dimer is an immunologic assay for which EDTA plasma should also be suitable, as it chelates added calcium, which is required to activate the functional assay. Correction for the dilution effect of liquid sodium citrate solution was suggested in a previous publication using heparin plasma as an alternative specimen [15] and was confirmed in this study for EDTA plasma for both D-dimer methods.

Among the strengths of this method comparison is the parallel, standardized measurement of freshly collected plasma samples with two different methods, as well as the gain of added value out of remnant material. The primary limitation is that selection was based on D-dimer results without clinical context. However, clinical information is neither mandatory for a method comparison nor a feasibility assessment. In addition, only two assays available on the market were included due to the limitation of available devices in our laboratory. Although the Atellica^®^ COAG 360 is no longer available on the market since summer 2024, and therefore, currently, no LOCI assay is available, the contemporary D-dimer assay can be used on other devices and could be established in a timely manner after verification.

We demonstrated that the use of EDTA instead of citrate plasma is feasible and might provide similar information. However, assuming the established cut-off of the contemporary assay for citrate plasma with 0.5 mg/L FEU was to be adopted, a clinical application of D-dimer measurement with EDTA instead of citrate plasma would not be readily applicable, even with volume correction. In a clinical setting, even a small difference in D-dimer levels may alter the work-up, either in the direction of further investigations being required to rule out a thromboembolic event or that it might be more challenging to dismiss other differential diagnoses. Accurate D-dimer measurement is a prerequisite for this, which is why a review of the cut-off value for both tests in EDTA plasma would be necessary for routine use in a clinical environment. This is additionally corroborated by the correlation and Passing–Bablok regression analysis close to the clinical decision value. Although our findings are promising, a verification with a larger number of samples near the clinical decision value in a prospective clinical setting is required before routine application. Apart from clinical situations, e.g., in research, D-dimer analysis in EDTA plasma proves to be a suitable substitute when citrated plasma is missing or in short supply.

## 5. Conclusions

Utilization of EDTA instead of citrate plasma is feasible and may provide similar diagnostic information. Despite excellent correlation, regression, and mean bias for both methods, the observed variance could have an impact on clinical interpretation and risk assessment. Apart from clinical situations, D-dimer analysis in EDTA plasma proves to be a suitable substitute. Therefore, further studies are required to confirm our results and, if necessary, determine cut-off values and clinical performance.

## Figures and Tables

**Table 1:** Precision assessment for INNOVANCE^®^ D-dimer with low (~0.25 mg/L fibrinogen equivalent units (FEU)) and high (~1.4 mg/L FEU) control plasma pool.

INNOVANCE^®^ D-Dimer	Grand Mean (±SD (Standard Deviation) (mg/L FEU))	Estimate for Repeatability (%CV (Coefficient of variation))	Estimate for Between-Run Precision (%CV)	Estimate for Within-Laboratory Precision (%CV)
Low (~0.25 mg/L FEU)				
Citrate	0.26 (0.012)	3.08	3.64	4.76
EDTA	0.25 (0.016)	6.27	1.22	6.38
High (~1.4 mg/L FEU)				
Citrate	1.43 (0.017)	1.04	0.68	1.24
EDTA	1.49 (0.024)	1.01	1.31	1.66

**Table 2:** Precision assessment for INNOVANCE^®^ LOCI high sensitivity (hs) D-dimer with low (~0.25 mg/L fibrinogen equivalent units (FEU)) and high (~1.4 mg/L FEU) control plasma pool.

INNOVANCE^®^ LOCI hs D-Dimer	Grand Mean (±SD (Standard Deviation) (mg/L FEU))	Estimate for Repeatability (%CV (Coefficient of variation))	Estimate for Between-Run Precision (%CV)	Estimate for Within-Laboratory Precision (%CV)
Low (~0.25 mg/L FEU)				
Citrate	0.23 (0.002)	0.75	0.54	0.93
EDTA	0.26 (0.003)	1.27	<0.00	1.27
High (~1.4 mg/L FEU)				
Citrate	1.35 (0.018)	1.19	0.65	1.35
EDTA	1.49 (0.030)	1.49	1.50	2.12

**Table 3:** Variance for each assay and specimen, as well as for cases <0.7 mg/L (n = 69) fibrinogen equivalent units (FEU). All quantitative values are given in mg/L FEU.

	D-Dimer Citrate Plasma	D-Dimer EDTA Plasma	D-Dimer EDTA-Corrected	hs D-Dimer Citrate Plasma	hs D-Dimer EDTA Plasma	hs D-Dimer EDTA-Corrected
Variance all (n = 99)	1.67	1.83	1.49	1.45	1.72	1.39
<0.7 mg/L (n = 69)	0.012	0.018	0.015	0.014	0.020	0.016

## Data Availability

The dataset can be requested from the authors upon reasonable request.
